# Chinese translation and psychometric testing of the cardiac self-efficacy scale in patients with coronary heart disease in mainland China

**DOI:** 10.1186/s12955-018-0872-4

**Published:** 2018-03-12

**Authors:** Xuelin Zhang, Yan Zhan, Jun Liu, Shouxia Chai, Lanlan Xu, Meirong Lei, Karen Wei Ling Koh, Ying Jiang, Wenru Wang

**Affiliations:** 1 School of Nursing, Hubei University of Medicine, Shiyan, Hubei China; 2 Department of Cardiovascular, Taihe Hospital, Hubei University of Medicine, Shiyan, Hubei China; 3 Department of General Surgery, Dongfeng Hospital, Hubei University of Medicine, Shiyan, Hubei China; 4 National University Heart Centre Singapore, National University Hospital, Singapore, Singapore; 5 Alice Lee Centre for Nursing Studies, Yong Loo Lin School of Medicine, National University of Singapore, Block MD 11, 10 Medical Drive, Singapore, Singapore

**Keywords:** Cardiac self-efficacy, Psychometric testing, Coronary heart disease, Chinese, Cross-cultural, Validation

## Abstract

**Background:**

A person’s self-efficacy plays a critical role during the chronic management process of a health condition. Assessment of self-efficacy for patients with heart diseases is essential for healthcare professionals to provide tailored interventions to help patient to manage the disease.

**Objective:**

To translate and test the psychometric properties of the Chinese version of Cardiac Self-efficacy Scale (C-CSES) as a disease-specific instrument for patients with coronary heart disease (CHD) in mainland China.

**Methods:**

The original English version of the CSES was translated into Chinese using a forward-backward translation approach. A convenience sample consisting of 224 Chinese patients with CHD were recruited from a university-affiliated hospital in Shiyan, China. The C-CSES and the General Self-efficacy Scale (GSES) were used in this study. The factor structure, convergent and discriminative validities, and internal consistency of the C-CSES were evaluated.

**Results:**

The confirmatory factor analysis (CFA) supported a three-factor high-order structure of the C-CSES with model fit indexes (RMSEA = 0.084, CFI = 0.954, NNFI = 0.927, IFI = 0.954 and χ ^2^ /df = 2.572). The C-CSES has good internal consistency with a Cronbach’s alpha of 0.926. The convergent validity of the C-CSES was established with significantly moderate correlations between the C-CSES and the Chinese version of the GSES (*p* < 0.001). The C-CSES has also shown good discriminative validity with significant differences of cardiac self-efficacy being found between patients with and without comorbidities of hypertension, diabetes, or heart failure.

**Conclusion:**

The empirical data supported that the C-CSES is a valid and reliable disease-specific instrument for assessing the self-efficacy of Chinese patients with CHD.

## Background

Coronary heart disease (CHD) remains one of the leading causes of death and disability among adults worldwide, and it has affected more than 10 million people in China [1]. Advancement in the treatment of CHD has resulted in improved survival. However, CHD patients are often confronted with an array of issues, such as lifestyle changes, management of medications, and uncertainty about the future [2, 3]. Cardiac self-efficacy (CSE) is a person’s confidence when he or she manages health outcomes imposed by his or her cardiovascular disease [4]. It has been reported that a person’s self-efficacy plays a critical role during the chronic management process of a health condition [5]. Studies have shown that CSE is an independent and strong predictor of quality of life and behavioral modification among patients with CHD [4, 6]. Lower CSE has been associated with poor coping strategies in dealing with CHD, unsatisfactory behavioral modification and poor health status, which lead to unexpected coronary events that result in a lower quality of life amongst patients with CHD [7, 8]. Thus, the assessment of CSE for patients with CHD will help health professionals to provide tailored interventions to enhance a patient’s self-efficacy, which will, in turn, help the patient to manage his or her disease effectively. However, there is lack of valid disease-specific measurement tools to assess the self-efficacy for Chinese-speaking patients with CHD.

The Cardiac Self-efficacy Scale (CSES) was developed as a cardiac-specific self-efficacy instrument, and its reliability and validity have been well established among patients with CHD [9]. The CSES has been increasingly utilized in assessing the cardiac self-efficacy in patients with CHD in different countries, such as Korea [10, 11], Australia [12], Iran [13], Sweden [14, 15], and Singapore [6, 16]. However, the CSES has only been validated in Sweden, where it has demonstrated good reliability and validity among patients with CHD [15]. With the approval from the author of the original CSES, our study was designed to translate the CSES into Chinese and evaluate the psychometric properties of the C-CSES among Chinese-speaking patients with CHD in mainland China.

## Methods

We conducted a two-phase study. In phase one, the English version of CSES was translated into Chinese. Its translation equivalence and content validity were examined. In phase two, the psychometric properties of the Chinese version of CSES (C-CSES) were tested. These properties included factor structure, convergent and discriminative validities, and the internal consistency of the C-CSES.

### Phase one: Translation and development of the C-CSES

After attaining permission from the authors of the original CSES [9], we followed a forward and backward translation approach based on Brislin’s Model of translation [17]. One bilingual author translated the CSES from the original language, English, into Chinese. A native Chinese speaker was then invited to do a monolingual review of the grammatical style and comprehensibility of the translated instrument to enhance the accuracy and understanding of the C-CSES [17]. Edits to the translated instrument based on feedback from the monolingual review were made before the back-translation. A second bilingual translator back-translated the instrument (Chinese to English) blindly.

Ten bilingual people were then invited to evaluate the translation equivalence of the two versions (English and Chinese) of the instrument. The translation equivalence of the CSES was evaluated using a 4-point scale (1 = not equivalent, 2 = somewhat equivalent, 3 = equivalent, and 4 = most equivalent). A scoring of 3 or 4 for all items would suggest a good translational equivalence of the C-CSES relative to the original version. Furthermore, a panel of seven experts (two cardiologists, four nurse specialists working in a cardiovascular department, and one nursing educator) was invited to review the translated CSES. The cultural relevance and content validity were evaluated using a 4-point rating scale ranging from 1 (not relevant) to 4 (very relevant). The content validity index (CVI), which is the percentage calculated based on the total items rated by the experts as either 3 or 4, was calculated.

### Phase two: Psychometric properties testing of the C-CSES

#### Sampling and data collection

Phase two of the study, which tested the psychometric properties of the C-CSES, was conducted on a convenience sample of Chinese patients with CHD at a university-affiliated hospital in Shiyan City, Hubei province, China from December 2015 to December 2016. Inclusion criteria were patients who (1) had a confirmed clinical diagnosis of CHD, (2) were able to read and understand Chinese, and (3) aged 18 years old or above. Those who had known major psychiatric disorders and other severe diseases (e.g., advanced cancer, end stage renal failure, etc.) were excluded.

The ratio of the number of subjects per item is an acceptable method to calculate the sample size needed to conduct factor analysis. Everitt [18] proposed that the minimum ratio of number of subjects per item should be 10:1. In this study, a ratio of 15 subjects per item was used to determine the sample size, and, accordingly, a total of 195 participants would be needed.

Ethics approval was obtained from the hospital’s ethics committee. All eligible patients were informed of the purpose and procedure of this study and their right to withdraw from the study at any time without affecting their treatment and nursing care. Their privacy was assured and maintained. Participants’ written informed consents were obtained.

#### Instruments

##### Cardiac self-efficacy scale (CSES)

The CSES was originally developed by Sullivan [9] and consisted of 13 items grouped into two subscales: control symptoms and maintain function. A 5-point Likert scale was used, ranging from 0 (not at all confident) to 4 (completely confident), with higher scores indicating a higher level of cardiac self-efficacy. The original English version of the CSES has demonstrated two-factor structure with a Cronbach’s alpha of 0.90 and 0.87 for the subscales: control symptoms and maintain function, respectively. However, the Sweden version of CSES presented a three-factor high-order structure after removing one unreliable item [15]. Both versions have shown good convergent and discriminant validities [9, 15].

##### General self-efficacy scale

The General Self-efficacy Scale (GSES) developed by Jerusalem and Schwarzer is a generic instrument used to measure self-efficacy in the nonclinical population [19]. It has been widely used to assess the self-efficacy of patients with various diseases [20–27]. The GSES has been translated into Chinese. It consists of 10 items, using a 4-point Likert scale from 1 (always false) to 4 (always true). With a unidimensional factor structure [28], the Chinese version of the GSES (C-GSES) has demonstrated good reliability with a Cronbach’s alpha of 0.91 [29].

Sociodemographic data such as age, gender, education level, employment status, and monthly income were self-reported by the participants, and clinical data such as blood pressure, body mass index (BMI), and comorbidities of hypertension, diabetes, and heart failure were collected from the hospital’s electronic medical records of the participants.

### Data analysis

SPSS version 24.0 and Amos 22.0 were used to analyze the data. The skewness and kurtosis were used to test the normality of C-CSES. Descriptive statistics, such as mean, standard deviation (SD), and frequency, were used to describe the participants’ characteristics. The percentage of participant’s highest and lowest possible scores of the total scale were used to examine the ceiling and floor effects of the C-CSES [30]. Confirmatory factor analysis (CFA) was performed with maximum-likelihood estimator to examine the best model fit of the C-CSES. The magnitude of the factor loadings were evaluated (> 0.71 excellent, > 0.63 very good, > 0.55 good, > 0.45 fair, and > 0.32 poor) [31]. Model fit evaluation was also assessed using the following index: root mean square error of approximation (RMSEA), comparative fit index (CFI), non-normed fit index (NNFI), incremental fit index (IFI), and chi-square/degree of freedom ratio (χ2/df). A model that shows an acceptable fit should have a χ2/df < 3, RMSEA < 0.08, CFI, NNFI and IFI > 0.9 [32]. The internal consistency of the C-CSES was tested using Cronbach’s α. The convergent validity of the C-CSES was examined using Pearson correlation to test the relationship between the C-CSES and the C-GSES. Discriminative validity of the C-CSES was tested using an independent t-test to compare the C-CSES scores between patients with and without comorbidities. A *p*-value of < 0.05 was considered to be of statistical significance.

## Results

### Translation equivalence and content validity

Based on the responses of the ten bilingual validators, the translation equivalence rate for overall C-CAES was 94.87% (ranged from 92.31% to 100%), indicating that the C-CSES correctly reflected the English version. The item CVIs ranged from 0.81–0.96, while the scale CVI was 0.87, indicating the good content validity of the C-CSES.

### Characteristics of subjects

A total of 285 patients with CHD were screened for eligibility in this study, of which, 254 (89.12%) met the study criteria and were enrolled into the phase two of the study. Of these participants, 224 (88.19%) completed the whole questionnaire and were included in the final data analysis.

The sociodemographic and clinical characteristics of the participants are presented in Table 1. The mean age of the participants was 58.87 (SD = 10.97) years old. The majority of them were male (*n* = 169, 75.45%) and lived with their families or relatives (*n* = 174, 77.68%). More than half of them (57.59%) had a secondary education, and one third of them (*n* = 74, 33.04%) were currently employed. The average BMI was 24.17 (SD = 4.00), and 57.14% of the participants were overweight. The means for systolic blood pressure and diastolic blood pressure were 124.16 mmHg (SD = 17.63) and 76.07 mmHg (SD = 10.80), respectively. In addition, more than half of them had comorbid hypertension, nearly one-third of the participants had comorbid diabetes, and 13.39% of the participants had comorbidity with heart failure.

**Table 1:** Sample characteristics (*n* = 224)

Characteristics	N	%
Gender		
Male	169	75.45
Female	55	24.55
Age(years)		
< 50	39	17.41
50–70	145	64.73
> 70	40	17.86
Living Status		
Living alone	50	22.32
Living with others	174	77.68
Employment Status		
Employed	74	33.04
Unemployed/ Retired	131	66.96
Education Level		
No formal/Primary education	56	25.00
Secondary education	129	57.59
Tertiary education	39	17.41
Co-morbidities		
Hypertension	115	51.34
Diabetes	83	37.05
Heart failure	30	13.39

Others including families, relatives and friends

### Item reduction

Fifty-two participants (20.47%) failed to answer item 12 “*maintain your sexual relationship with your spouse.*” Further analysis indicated that there was a significant difference in the mean age between those who responded to item 12 (mean age = 57.71 ± 10.65) and those who did not (mean age = 67.69 ± 9.38) (*P* < 0.001). This would be a threat to the validity of the C-CSES. Therefore, item 12 was removed from the C-CSES.

### The descriptive statistics of C-CSES

The normality of each C-CSES item was assessed based on the values of skewness and kurtosis. The range of skewness was between 0.029 and 0.369, and the range of kurtosis was between 0.140 and 0.790, indicating that the items of the C-CSES were normally distributed (Table 2). The mean score of the C-CSES total was 27.54 (SD = 9.63), while the mean scores of individual items ranged from 2.11 to 2.50 (SD = 0.80–1.17). Among all the participants, 0.47% scored the highest scores for the total scales, indicating low ceiling effects of the total scale. There were no participants who scored the lowest scores, indicating no floor effects of the total scale.

**Table 2:** Mean, standard deviations, and skewness and kurtosis of C-CSES (*n* = 224)

Items	Mean	SD	Skewness	Kurtosis
1. When you should call or visit your doctor about your heart disease	2.18	1.17	−.047	−.790
2. How to make your doctor understand your concerns about your heart	2.11	0.95	−.131	−.234
3. How to take your cardiac medications	2.39	1.06	−.197	−.711
4. How much physical activity is good for you	2.19	0.80	.236	−.140
5. Control your chest pain by changing your activity levels	2.14	0.96	−.103	−.238
6. Control your chest pain by taking your medications	2.17	0.89	.030	−.234
7. Control your breathlessness by changing your activity levels	2.14	0.94	−.029	−.568
8. Control your breathlessness by taking your medication	2.17	0.89	−.198	−.573
9. Maintain your usual social activities	2.40	1.06	−.369	−.422
10. Maintain your usual activities at home with your family	2.50	0.98	−.320	−.401
11. Maintain your usual activites at work	2.40	0.98	−.336	−.352
13. Get regular aerobic exercise (work up a sweat and increase your heart rate)	2.26	1.02	−.214	−.380

*C-CSES* Chinese version of Cardiac Self-efficacy Scale, *SD* Standard deviation

### Factor structure and internal consistency of the C-CSES

**Fig. 1:**
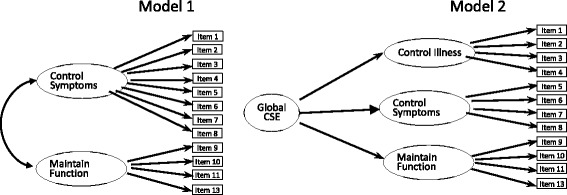
Factor structures of models 1–2. CSE: cardiac self-efficacy

CFA was performed to confirm the underlying factor structure of the C-CSES, using AMOS 22.0 (Fig. 1). Initially, a two-factor model (Fig. 1, Model 1) for the C-CSES was applied to the 224 participants to test the validity of factor structure according to the original study conducted in the United States [9], however the model fit was unacceptable (RMSEA = 0.124, CFI = 0.885, NNFI = 0.87, IFI = 0.896, χ^2^/df = 4.42) (Table 3).

**Table 3:** Comparison of C-CSES models (*n* = 224)

Model	RMSEA	CFI	NNFI	IFI	χ2/df
Model 1: Two factor model	0.124	0.885	0.87	0.896	4.42
Model 2: Three factor model	0.084	0.954	0.927	0.954	2.572
Model 3: Three factor model and covariance between the residuals of item1 and 2	0.064	0.974	0.947	0.966	1.899

*C-CSES* Chinese version of Cardiac Self-efficacy Scale

**Fig. 2:**
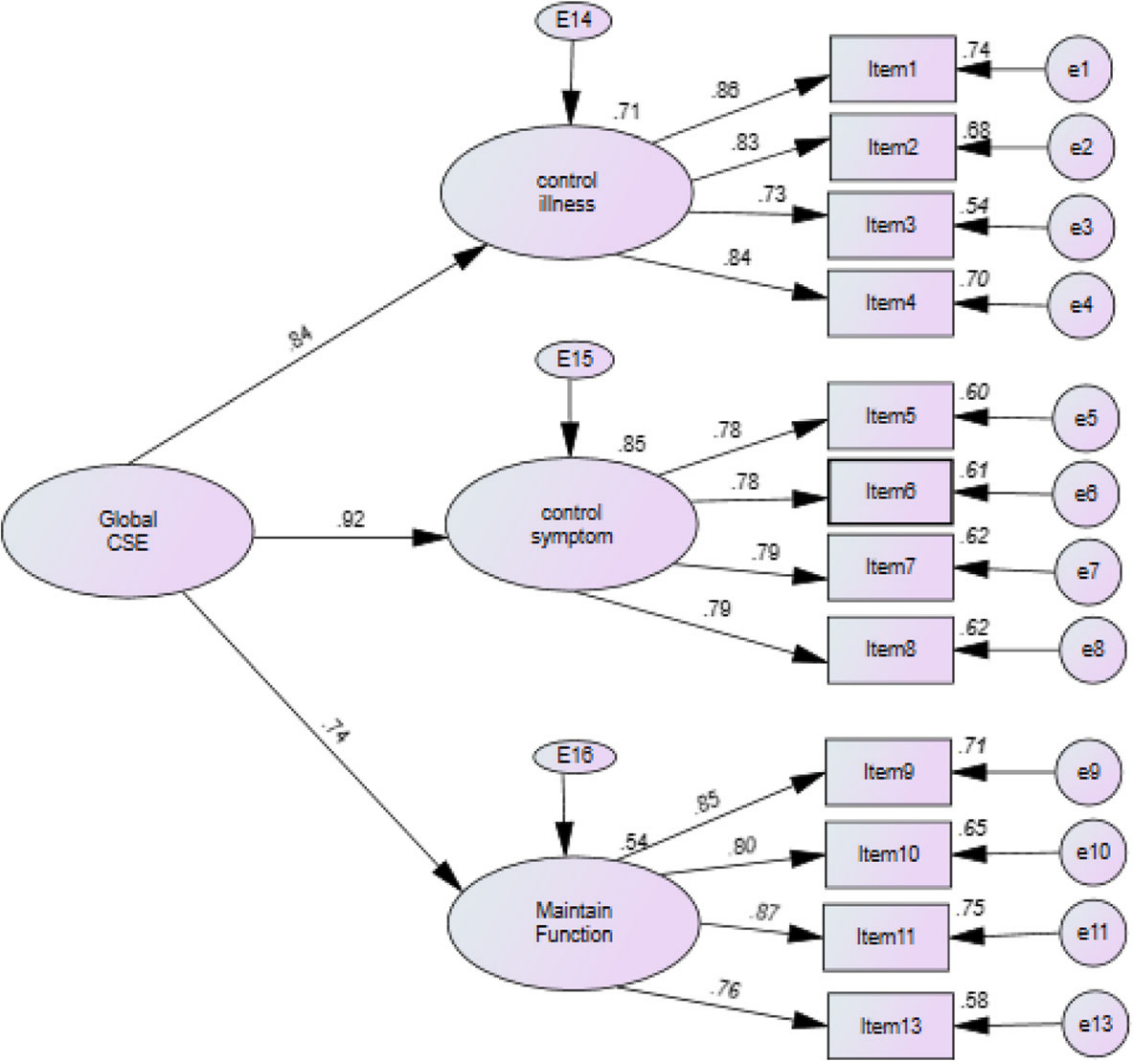
Factor loading of the second-order factor model (Model 2). CSE: cardiac self-efficacy

Then, a three-factor high-order model (Fig. 1, Model 2) based on the Swedish validation study [15] was investigated to further test the validity of factor structure, wherein the first eight items were divide into two dimensions ‘control symptoms and control illness’ equally, last four items were in the dimension of ‘maintain function’, the global factor was drawn from the three factors. It was shown that the standardized factor loadings of all items were statistically significantly and positively correlated to each subscale, the factor loadings on the global factor of cardiac self-efficacy were also generally high (Fig. 2), the model fit was RMSEA = 0.084, CFI = 0.954, NNFI = 0.927, IFI = 0.954, and χ^2^/df = 2.572 (Table 3). Modification indices of this model showed a relatively strong covariance of the residuals between items 1 and 2. If taking this covariance into consideration, the model fit would be improved (χ^2^ = 94.963, χ^2^/df = 1.899).

Though the RMSEA of model is slight over than 0.08, it is the better model fit for C-CSES. The model 2 was selected to be the final model for the C-CSES. The factor structure for the model is illustrated in Fig. 2. The internal consistency of the C-CSES was good with a Cronbach’s α of 0.926 for the total scale.

### Convergent and discriminative validities

There was a significant and moderate correlation between the C-CSES and the C-GSES (*r* = 0.470, *p* < 0.01), indicating a good convergent validity of the C-CSES. In addition, the discriminative validity of the C-CSES was demonstrated. There were significant differences of the C-CSES total and subscales between patients with and without comorbidities. Patients with a comorbidity of hypertension had significantly lower scores of the C-CSES total and three subscales than those without hypertension (*p* < 0.01). Patients with a comorbidity of diabetes had significantly lower scores of total and subscales than those without diabetes (*p* < 0.001), while the patients with a comorbidity of heart failure reported significantly lower scores in all subscales than those without heart failure (*p* < 0.001) (Table 4).

**Table 4:** Comparison of scale means of C-CSES by comorbidities (*n* = 224)

Variables	Control Illness	Control Symptoms	Maintain Function	Total
Comorbid with hypertension				
Yes (*n* = 115)	8.29(3.57)	8.03(3.47)	8.81(3.54)	25.13(9.41)
No (*n* = 99)	9.55(3.43)	9.30(3.25)	10.3593.30)	29.20(8.70)
*t*	2.67	2.82	3.36	3.35
*p value*	0.008**	0.005**	0.001**	0.001**
Comorbid with diabetes				
Yes (*n* = 83)	7.67(3.14)	7.37(3.25)	7.51(3.16)	22.55(8.33)
No (*n* = 141)	9.62(3.60)	9.40(3.30)	10.77(3.12)	29.79(8.77)
*t*	4.1	4.47	7.51	6.08
*p value*	0.000**	0.000**	0.000**	0.000**
Comorbid with heart failure				
Yes (*n* = 31)	6.69(2.66)	6.16(2.75)	7.12(2.94)	20.19(6.94)
No (*n* = 193)	9.22(3.58)	9.05(3.35)	9.94(3.44)	28.22(9.14)
*t*	4.27	4.559	4.318	4.675
*p value*	0.000**	0.000**	0.000**	0.000**

*t* Independent t-test, *F* Analysis of variance, *C-CSES* Chinese version of Cardiac Self-efficacy Scale

**p < 0.01

## Discussion

Given the wide range of cultural and social differences between Chinese-speaking and English-speaking patients [33] and that the cross-cultural use of the CSES is common [6, 10, 11, 16], translation and cross-cultural validation of the original CSES are important. The current study followed the standard forward-backward translation process to evaluate the psychometric properties of the Chinese version of CSES in Chinese patients with CHD. The results of our study indicated that the C-CSES has good semantic equivalence and content validity, which is comparable with the Sweden version tested with CHD [15].

Item 12 “*maintain your sexual relationship with your spouse”* was removed from the final C-CSES because 20.47% participants failed to respond to it. Those who failed to respond were older patients. Compared to the younger generation, older Chinese are more conservative and traditional. Most of them consider a sexual relationship with a partner as a private matter and do not wish to discuss it publicly [34]. Therefore, this item was not suitable for this population. It is consistent with the validation study of the Chinese cardiac depression scale, in which the item related to sexual activity was excluded because of poor cultural relevance [35].

The factorial structure of the C-CSES identified from the CFA was similar to the Swedish version of the CSES. In particular, items loading on the dimension of control symptom were found to be fully in line with the Swedish study. However, items loading on the other two dimensions were different. Such difference could be due to the item deduction in a different cultural environment. In the study conducted in Sweden [15], the item “*How much physical activity is good for you*” was considered unreliable, and, therefore, removed from the Swedish version of the scale. This resulted in one less item loading on the dimension of control illness compared to the current study. Similarly, in the current study, the item “*maintain your sexual relationship with your spouse*” was considered as culturally irrelevant and removed from the C-CSES. This item reduction resulted in one less item loading on the dimension of maintain function when compared to the Sweden version. The result of this study is inconsistent with the original version of the CSES [9], in which a two-factor structure model was reported [9]. The inconsistent models reported in different languages might be because patients from different social and culture background may perceive their confidence in controlling illness and symptoms differently. Nevertheless, our study results revealed the best model fit with the Swedish version of the CSES and affirmed the three higher-order factor structure of the C-CSES.

Previous studies had reported that the presence of comorbidities, such as hypertension, diabetes, and heart failure, significantly impaired patients’ self-efficacy and health outcomes [35–38]. The number of comorbidities was negatively associated with patients’ level of self-care ability and quality of life [35–38]. The discriminative validity of the C-CSES was confirmed in our study, which were that significant differences of the C-CSES were found between the patients with and without comorbidities.

The internal consistency of the C-CSES was satisfied with a Cronbach’s α of 0.926 for the total scales. This finding corresponded well with those reported in the original English [9] and Swedish versions [15]. The current study was the first to test the floor and ceiling effects of the CSES. Based on the results, the C-CSES has a low ceiling without floor effects for the total scale. The C-CSES also demonstrated good convergent validity with significantly moderate correlations with the generic C-GSES. This is consistent with the previous validation study conducted in Sweden [15]. Compared to the C-GSES, the C-CSES is a disease-specific instrument; therefore, the scale would have better specificity in measuring the self-efficacy among patients with heart disease in China.

### Limitation

We acknowledge that this study has several limitations. First, the study participants were recruited from one university-affiliated hospital in southern China, which may make it difficult to generalize the findings to a wider population in China. Second, the stability of the C-CSES is not confirmed, as we did not perform test-retest reliability in the current study. Future study to examine the stability of the C-CSES is recommended.

## Conclusions

Our study results confirmed a three-factor high-order model of the C-CSES with the best model fit, and it has good internal consistency and satisfied convergent and discriminative validities. It can be used by healthcare professionals as a valid and reliable disease-specific tool to assess the self-efficacy in Chinese-speaking patients with CHD.

## Abbreviations

BMI: Body mass index
C-CSES: Chinese version of Cardiac Self-efficacy Scale
CFA: Confirmatory factor analysis
C-GSES: Chinese version of General Self-efficacy Scale
CHD: Coronary heart disease
CSES: Cardiac Self-efficacy Scale
CVI: Content validity index
GSES: General Self-efficacy Scale
